# Protein Kinase CK2: A Targetable BCR-ABL Partner in Philadelphia Positive Leukemias

**DOI:** 10.1155/2015/612567

**Published:** 2015-12-30

**Authors:** Alessandro Morotti, Giovanna Carrà, Cristina Panuzzo, Sabrina Crivellaro, Riccardo Taulli, Angelo Guerrasio, Giuseppe Saglio

**Affiliations:** ^1^Department of Clinical and Biological Sciences, University of Turin, 10043 Orbassano, Italy; ^2^Department of Oncology, University of Turin, 10043 Orbassano, Italy

## Abstract

BCR-ABL-mediated leukemias, either Chronic Myeloid Leukemia (CML) or Philadelphia positive Acute Lymphoblastic Leukemia (ALL), are the paradigm of targeted molecular therapy of cancer due to the impressive clinical responses obtained with BCR-ABL specific tyrosine kinase inhibitors (TKIs). However, BCR-ABL TKIs do not allow completely eradicating both CML and ALL. Furthermore, ALL therapy is associated with much worse responses to TKIs than those observed in CML. The identification of additional pathways that mediate BCR-ABL leukemogenesis is indeed mandatory to achieve synthetic lethality together with TKI. Here, we review the role of BCR-ABL/protein kinase CK2 interaction in BCR-ABL leukemias, with potentially relevant implications for therapy.

## 1. Introduction

The t(9;22) chromosomal translocation (also known as Philadelphia chromosome, or Ph^+^) is the genetic hallmark of Chronic Myeloid Leukemia (CML) and characterizes one quarter of adult Acute Lymphoblastic Leukemia (ALL) and less than 5% of pediatric ALL [1–4]. CML is sustained by the p210-BCR-ABL isoform, while Ph^+^-ALL is driven by a shorter p190-BCR-ABL isoform [5]. BCR-ABL leukemias are the paradigm of cancer targeted therapy, due to the successful development of BCR-ABL specific tyrosine kinase inhibitors (TKIs). However, CML and Ph^+^-ALL still challenge clinicians and biologists. Clinicians are facing the fact that CML is still an uncurable disease [6]. Even if CML is effectively targeted by TKIs with astonishing responses rates, most of those patients that discontinue TKI therapy eventually relapse [7] due to the persistence of TKI resistant CML stem cells [8–11]. The second, Ph^+^-ALL, is associated with much worse responses to TKI than those observed in CML [1, 12] and therefore requires additional targets to achieve synthetic lethality together with TKI. Furthermore, clinicians have also to address the issue of TKI resistance due to the development of BCR-ABL point mutations [13]. Therefore, the definition of those pathways that are necessary for the maintenance of Ph^+^ leukemias could identify novel targets to achieve synthetic lethality together with TKI. Here, we will review the role of protein kinase CK2 (Casein Kinase 2, CK2 from here on) as a BCR-ABL substrate.

## 2. Protein Kinase CK2

### 2.1. Biological Characterization

CK2 is an ubiquitously expressed serine-threonine kinase [14–21]. It is composed of two catalytic and two regulator subunits. The catalytic units are represented by the isoforms CK2*α* and CK2*α*′; the regulatory unit is composed of the CK2*β*isoform. Each subunit is the product of different genes. The relevance of CK2 in biological processes is highlighted by the phenotype of CK2 knockout mice. In particular, both CK2*α* [22, 23] and CK2*β* [24] knockout mice are lethal with multiple embryonic alterations; however, knockout mice of CK2*α*′ are viable [25], although sterile, suggesting some grade of compensations among the CK2 catalytic subunits. The CK2*β* subunit is highly conserved among species and is involved in the assembly of the tetrameric complex with the catalytic subunits and in the modulation of substrate recognition. Two CK2*β* interact with two identical (two CK2*α* or two CK2*α*′) or nonidentical (one CK2*α* and one CK2*α*′) catalytic subunits. The CK2 kinase is able to phosphorylate serine or threonine residues in proteins bearing a minimal consensus sequence that contains an acidic residue (Glu, Asp, pSer, or pTyr). CK2 is a unique kinase in that it can utilize GTP as well as ATP as the phosphate donor [19]. CK2 was often referred to as a constitutively active kinase, although several reports suggested different chances of kinase modulation [26]. In particular, it was extensively reviewed that CK2 activity can be modulated by changes in the subunit assembly, interaction with different regulatory elements, and protein interaction and finally even through different levels of phosphorylation/autophosphorylation [16, 19, 26, 27]. Several phosphorylation residues have indeed been identified both in the catalytic and regulatory subunits. Even if these phosphorylation sites did not appear to directly affect the kinase activity, these sites could affect the stability of the tetramer and therefore regulate substrate phosphorylation.

### 2.2. CK2 Targets

**Figure 1:**
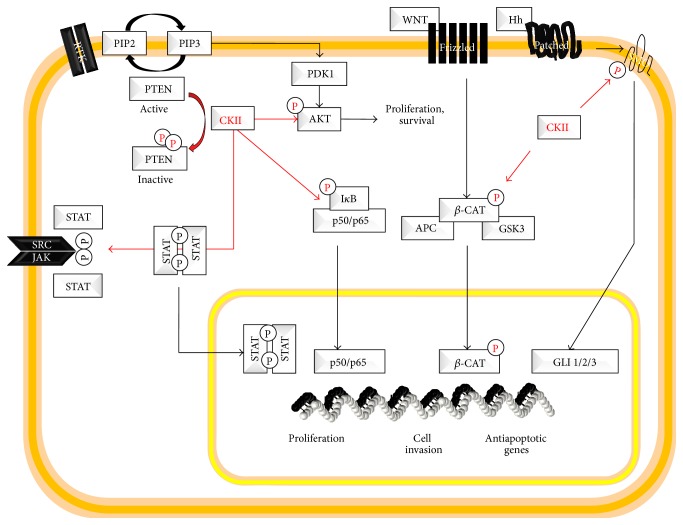
Protein kinase CK2 targets. This figure summarizes major CK2 targets.

Beside the complex mechanisms of CK2 regulation and activation, which still require further investigations, it is well documented that CK2 phosphorylates several different targets, as extensively reviewed [18, 28]. CK2 was discovered in each cellular compartment, from the membrane to the nucleus, suggesting that it can interact and regulate the function of several proteins in every cellular compartment. In particular, CK2 is known to regulate cellular proliferation and apoptosis, DNA damage repair and gene expression, regulation of cell structure, and other cellular processes.  Figure 1 shows some of the cancer associated targets, such as AKT, IkB-*α*, STAT5, and *β*-catenin.

### 2.3. CK2 Inactivates PTEN

PTEN is a tumor suppressor that negatively regulates the PI3K-AKT pathway, therefore counteracting one of the major signaling transduction networks involved in cancer pathogenesis [29, 30]. PTEN function is regulated by several posttransductional modifications such as serine/threonine-phosphorylation, acetylation, ubiquitination, and sumoylation [30]. Notably, the C-terminal domain of PTEN contains six serine/threonine residues (Thr-366; Ser-370; Ser-380; Thr-382; Thr-383 and Ser-385) that regulate the activity of the phosphatase PTEN, cellular compartmentalization, and protein stability [31–35]. Even if Ser-370 and Ser-385 were identified by mass-spectrometry as the mostly phosphorylated sites in PTEN [36, 37], all these residues have been described as CK2 substrates. Notably, CK2-mediated PTEN tail phosphorylation was clearly shown to play a role in different Philadelphia chromosome negative leukemias [38–42].

## 3. CK2 Inhibitors

Several CK2 inhibitors have been developed with different grades of selectivity and potency, as extensively reported [17, 43–54]. CK2 inhibitors have already been tested in hematological cancers. In particular, Chronic Lymphocytic Leukemia has been extensively studied for its high sensitiveness to CK2 inhibitors [41, 55]. Similarly, CK2 inhibitors appeared to display important effects in T-ALL [56]. Currently, some clinical trials are ongoing and will assess the relevance of CK2 inhibitors in the setting of hematological and solid cancers.

## 4. CK2 in Philadelphia Positive Leukemias

**Figure 2:**
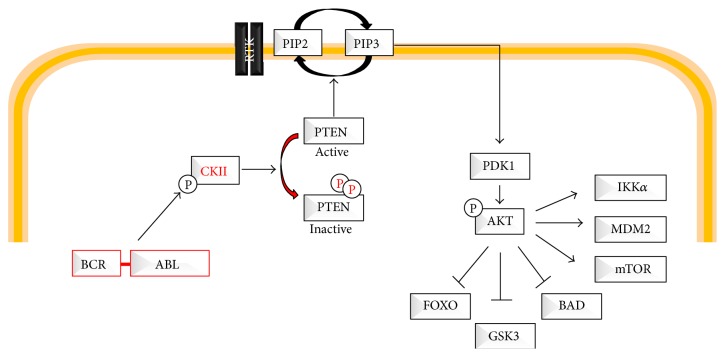
BCR-ABL/CK2/PTEN pathway. This figure describes the BCR-ABL/CK2/PTEN pathway.

An original report showed that CK2 is highly expressed in proliferating CML myeloid progenitors [57]. Later, it was shown that BCR-ABL is able to physically interact with CK2*α* in K562 cell line, via the ABL portion of the chimeric protein [58]. Similarly, CK2*α* was shown to interact with c-Abl in NIH3T3 cells. Furthermore, BCR-ABL appeared to phosphorylate CK2*α* on tyrosine residues. Notably, in this first report, BCR-ABL was shown to inhibit the function of CK2*α* [58]. BCR-ABL/CK2*α* interaction was also investigated by another group [59], who demonstrated that CK2*α* strongly interacts with the BCR region between amino acids 242 and 413. Oppositely to the first report, CK2*α* was shown to positively mediate BCR-ABL signaling in both CML and Ph^+^-ALL [59]. Treatment with CK2*α* inhibitor 4,5,6,7-tetrabromo-2-benzotiazole was indeed shown to inhibit the growth of both p210- and p190-BCR-ABL transformed cells and BCR-ABL positive cells. Notably, the inhibition of BCR-ABL with TKI is also associated with the reduction of CK2*α* serine/threonine kinase activity. These original observations offer important implications for the therapeutical approach of BCR-ABL-positive CML/ALL and for the definition of CK2 regulation mechanisms. In particular, while CK2 has always been referred to as a constitutively active kinase, this work demonstrated that BCR-ABL regulates CK2 kinase activity, even if through a complex yet unknown mechanism. Another report further highlights the utility of targeting CK2 in the setting of BCR-ABL-mediated leukemias and in particular p190-BCR-ABL ALL cells [60]. A great step forward in the understanding of the role of BCR-ABL/CK2 complex was carried out by the group of Donella-Deana [61]. In particular, while the first two reports lead to opposite conclusions, probably due to different cellular context, the last report confirmed that BCR-ABL interacts with CK2*α* in CML cells and that this interaction promotes cellular proliferation. Furthermore, this work provided additional insights on the mechanisms of CK2*α* regulation by BCR-ABL. In particular, authors have shown that CK2, both CK2*α* and CK2*β*, may be upregulated in imatinib-resistance CML cell lines with a consequent increase in the CK2 kinase activity [61]. Notably, no changes on the mRNA levels were observed, clearly suggesting upstream CK2 regulation. This observation, and the previous observation that CK2*α* tyrosine phosphorylation by BCR-ABL affects the CK2 kinase activity, suggests that in CML CK2 not only is just a constitutively active kinase but also can be somehow regulated. To further investigate the mechanisms of interaction, authors have also confirmed that BCR-ABL tyrosine phosphorylates CK2 and that this event is not required for the interaction between the two kinases. However, inhibition of CK2 abrogates the interaction. Although these data did not shed light on the complex mechanism of CK2 regulation, it is clear that BCR-ABL is able to force CK2 to modulate proliferation/survival in Ph^+^ leukemias. The authors have indeed clearly confirmed that CK2 inhibitor CX-4945 is able to promote cell death [61]. All these works did not link BCR-ABL/CK2*α* interaction with specific CK2*α* targets [58–60]. Recently, we have demonstrated that BCR-ABL/CK2*α* promotes serine phosphorylation of PTEN tail [62] (Figure 2). PTEN is found mostly in the cytosol of CML progenitor cells [63] where it is highly phosphorylated by CK2. PTEN tail phosphorylation inhibits its phosphatase activity both in cellular models and in primary CML cells. Interestingly, PTEN mutants, unable to be phosphorylated by CK2*α*, restored the phosphatase activity and were able to promote strong apoptosis induction in CML cells. Altogether, these works demonstrate that BCR-ABL interacts with CK2*α* which is in turn tyrosine phosphorylated [58, 59, 61]; BCR-ABL somehow “activates” CK2*α* towards substrates that are involved in the regulation of proliferation and survival. Lastly, BCR-ABL/CK2*α* interaction promotes the phosphorylation of PTEN with consequent inactivation of its phosphatase activity [62].

## 5. Conclusions

Since the discovery of the t(9;22) translocation, the Philadelphia chromosome, as the hallmark of CML, this disease has been the paradigm of precision medicine. However, BCR-ABL targeting with TKI did not allow eradicating both CML and Ph^+^-ALL, therefore highlighting the need of combinational therapies. In this review, we have summarized the role of CK2 as an essential mediator of BCR-ABL oncogenic signal. The BCR-ABL/CK2 complex is indeed responsible for mediating BCR-ABL induced cellular proliferation and survival. Targeting CK2 with specific inhibitors has been clearly shown to achieve synthetic lethality together with TKI, suggesting that a combinatorial therapy could help in eradicating Ph^+^ leukemias. Finally, the intriguing role of BCR-ABL/CK2 complex as being able to functionally inactivate the tumor suppressor PTEN may point to a highly effective proapoptotic therapy even in those cases characterized by TKI resistance due to BCR-ABL mutations.
